# Research on directional rock blasting based on different slotted pipe materials of the combined charge structure

**DOI:** 10.1038/s41598-024-57957-4

**Published:** 2024-03-28

**Authors:** Lianhua Wu, Yiping Zhang, Tianliang Hou, Kaixin Liu, Yusong Miao, Jie Li, Xin Zhao, Mei Zhang

**Affiliations:** 1 Mining College, Guizhou University, Guiyang, 550025 China; 2 Guizhou Polytechnic of Construction, Guiyang, 551400 China; 3 School of Science, Qingdao University of Technology, Qingdao, 266520 China; 4 China Railway 19th Bureau Group Mining Investment Co., Ltd., Beijing, 100161 China; 5 Xuanhua Vocational College of Science & Technology, Zhangjiakou, 075100 China

**Keywords:** New charge structure, Shaped charge blasting, Slotted cartridge, Model test, Stress–strain analysis, Blast-generated crack distribution, Civil engineering, Energy infrastructure, Engineering

## Abstract

For shaped charge blasting projects in mining, civil engineering, and similar fields, it is proposed to modify the charge structure by combining slotted tubes and shaped charge liners to obtain a new type of charge structure. This aims to achieve directional rock breaking through the focused action of the shaped charge. The influence of different slotted pipe materials on the directional rock-breaking effect of concentrated energy using a new charge structure is explored through theoretical analysis combined with model test study, high-speed camera, stress–strain gauge, and other equipment. A comparison is made between slotted pipes made of aluminum, kraft paper, and PVC, with the cutting width of 2 mm. Based on the characteristics of the cracks formed after blasting, the new charge structure made of aluminum slotted pipe produces a penetrating crack that is almost consistent with the pre-cracking direction. Based on the corresponding characteristics of successively released blasting energy, the guiding and convergence effect of the new charge structure made of aluminum slotted pipe on the explosion energy is greater than that of the new charge structure made of the other two types of slotted pipe material. According to the strain data measured after blasting, the peak arrival time of the strain peak in the direction of the slotted pipe on one side of the shaped hood is shorter than that in the other two directions, and the peak strain is greater than that in the other two directions while having a better energy gathering effect. Based on the findings, the new charge structure with directional energy concentration has a damage reduction effect. Furthermore, the material of aluminum slotted pipe is found to be better than PVC slotted pipe, whereas the material of PVC slotted pipe is better than kraft paper slotted pipe in achieving directional rock breaking.

## Introduction

The drilling-blasting method^1,2^ can harm the surrounding rock, compromising the stability of the rock mass^3,4^. Techniques like smooth-surface charge blasting and directional blasting can mitigate this damage but face challenges like overblasting, underblasting, preserving the rock mass, and blasting vibration. Enhancing the directional effect of blasting charges is crucial to solving these problems.

Advances in understanding rock blasting mechanisms have been made through theoretical analysis^5–7^, experiments^8,9^ and numerical simulations^10–15^.Huo et al. ^16^ established a numerical model of three-row boreholes and optimized the charge structure for damage control of large-aperture lateral blasting excavation rock. Ge et al. ^17^ studied the jet formation behavior and optimization of trunconical hypercumulation shaped charge structure, revealing its advantages over the traditional conical-shaped charge structure. Wang et al. ^18^ used the numerical analysis technique to study the directional fracture mechanism of the slit drug pack and analyzed the directional fracture effect under different charge structures. He et al.^19^ conducted single- and double-hole blast loading tests on granite samples to investigate the dynamic failure process between adjacent boreholes when using decoupling charges. Yin et al. ^20^ reported that the optimum blasting and fracturing effect of the surrounding rock mass can be achieved by uncoupled-shaped charge technology. Zhu et al. ^21–23^ investigated the influence of the uncoupled charge coefficient on stress distribution and main fracture propagation in rock mass. Man et al.^24^ analyzed the spectrum of blasting vibration signal and wavelet packet of different charge structures in the surrounding holes and found that the maximum blasting vibration velocity of the slit pack was lower than that of the ordinary pack. Xia et al. ^25^ analyzed the effect of charge structure on energy transfer and revealed that the transfer of explosive energy was affected by the decoupling charge coefficient between the explosive and the rock, and different charge structures exhibited different energy utilization rates.

In terms of the constraints of the medicine bag, Fourney et al. ^26^ made symmetrical bilateral incisions on the tubular medicine bag and found that the medicine bag with a slit showed enhanced directional fracture effect. Yang et al. ^27,28^ studied the propagation characteristics of the explosion shock wave for the seams with different materials such as stainless steel pipe, PVC pipe, and plexiglass pipe. They found that the expansion forms of the explosion shock wave and the explosion gas for the three materials were the same while maintaining a highly symmetrical form and propagating from the cutting pipe to the outside without being affected by the material of the cutting pipe. Yang et al.^29^ employed the orthogonal design method to optimize the wall thickness, slot width, slot length, and decoupling coefficient of slotted cartridges in blasthole. They determined appropriate structural parameters for the industrial production of the slotted pipe and verified the directional effect of the slotted direction through the induced detonation test. Zuo et al. ^30^ investigated the filling mediums of air, sand, water, and a solid–liquid mixture (sand and water) positioned between the explosive and the hole wall. They established a three-dimensional reconstruction model of "rock-explosion cracks" under various filling mediums. Similarly, Zhang et al.^31^ examined the damage to concrete specimens using different filling mediums. In addition, several researchers have explored the mechanism, propagation law, and energy utilization rate of pre-existing cracks through various methods and influencing factors^32–35^. Yua^36^, drawing on the thick-walled cylinder theory and fracture mechanics theory, explored the crack formation mechanism of pre-splitting blasting. They also developed the criteria for the initiation, propagation, and termination of initial cracks under the impact of a shock wave. Luo et al.^37^ analyzed the propagation law of 0°, 30°, 60°, and 90°pre-split on the primary crack of grooving blasting. They found that with an increase in the pre-splitting angle, the crack propagation complexity of the main crack initially decreases and subsequently increases.

These studies have contributed substantially to understanding the shaped energy blasting mechanism and reducing surrounding rock damage. However, the material and charge structure of the slotted pipe still offer vast potential for further research. This research introduces a novel charge structure that combines a slotted pipe and a shaped hood. It employs theoretical analysis and model testing to examine the impact of different slotted pipe materials on the pre-splitting blasting effects of the new charge. The findings have noteworthy implications for enhancing the effect of slotted cartridges.

## Directional rock-blasting mechanism of explosion energy of new charge structure

Blasted rocks result from the interplay of blast shock waves and quasi-static pressures ^34^. Due to the exceedingly brief reaction time of the blasted rock and the varied properties of the blasted rock mass, quantitatively characterizing the action process and features during the research process becomes challenging. This complexity has made the study of the blasting rock mechanism intricate. The following discusses the theories of rock breaking for slotted cartridge blasting and shaped charge blasting, respectively. Subsequently, the rock-breaking mechanism of the new charge structure is analyzed.

### Theoretical analysis of rock breaking by slotted cartridge blasting

**Figure 1:**
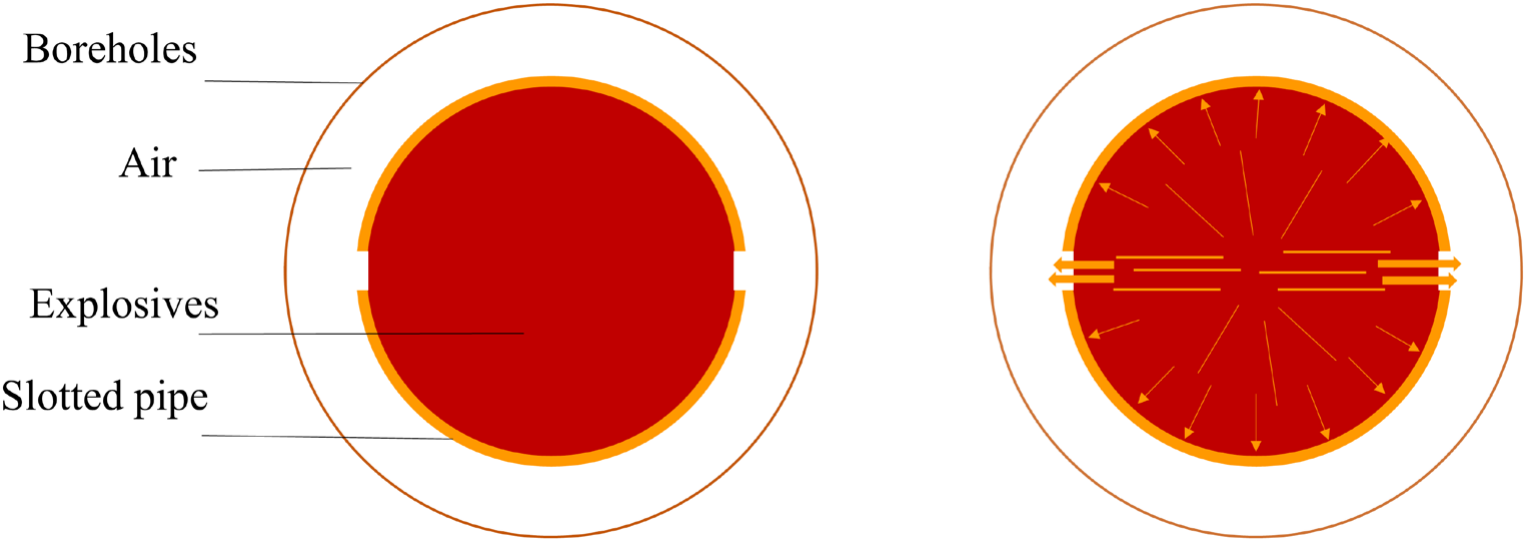
Slotted cartridge blasting model.

Slotted cartridge blasting involves creating slits of various angles, shapes, and quantities on the shell of an explosive of a specific density and strength^38^ (Fig. 1). The slotted cartridge directs the formation of blasting directional fracture cracks, which generally occur in two phases: the initial directional crack formation and the propagation and extension of these cracks. The stress intensity factor at the crack tip during the propagation phase is detailed in ^39^:

1 $$ K_{I} = pF\sqrt {\pi \left( {R_{a} + a} \right)} $$

where a is the fracture length; ${R}_{a}$ is the borehole radius; F is the stress intensity factor correction factor, i.e., $F=F[{(R}_{a}+a)/{R}_{a}]$.

When ${K}_{I}\ge {K}_{ID}$ (${K}_{ID}$ is the rock fracture toughness), the fracture will continue to start cracking, then the pressure in the borehole is ${p}_{0}$ at this time, then ${p}_{0}$ must satisfy the following conditions^39^:

2 $$ p_{0} \ge \frac{{K_{ID} }}{{F\sqrt {\pi (R_{a} + a)} }} $$

where ${a}_{0}$ is the depth of the cut formed by the jet.

### Theoretical analysis of shaped charge blasting

**Figure 2:**
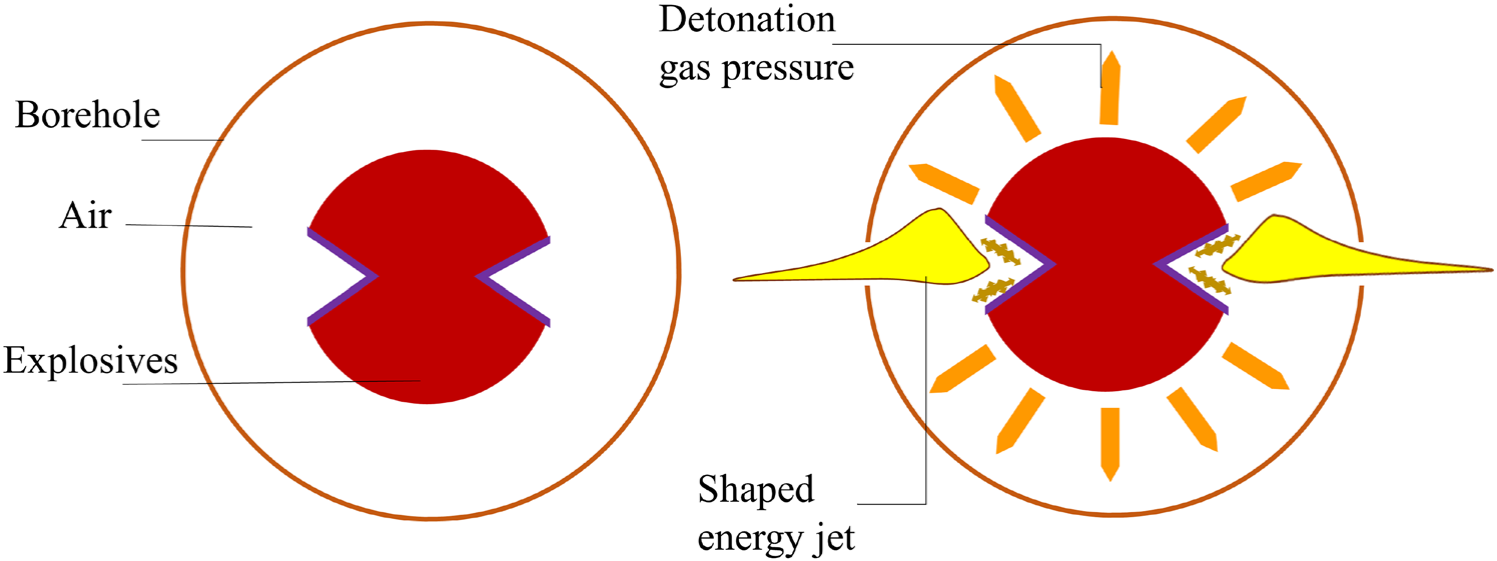
Shaped charge blasting model.

Shaped charge blasting incorporates a hole at one end of the pack to enhance localized damage. A specially shaped pillar is utilized to focus the explosion products and augment the explosive energy flow density, thereby amplifying the explosive force. Figure 2 depicts a model of a shaped charge blast. The stress intensity factor at the fracture tip is addressed during the blast crack expansion.

During the blast crack expansion, the stress intensity factor at the fracture tip is:

3 $$ K_{I} = PF\sqrt {\pi (r_{b} + a)} + \sigma_{\theta } \sqrt {\pi a} $$

where P is the burst gas pressure (MPA); F is the fracture tip stress intensity factor correction factor; ${r}_{b}$ is the radius of the borehole (m); a is the fracture length (m); ${\sigma }_{\theta }$ is the tangential stress (MPa).

Based on fracture mechanics theory, when the stress intensity factor at the fracture tip exceeds the rock's fracture toughness, the crack starts to crack and expand. Therefore, the burst gas pressure required to meet the continuous expansion of the blast fracture is ^35^:

4 $$ P > \frac{{K_{IC} - \sigma_{\theta } \sqrt {\pi a} }}{{F\sqrt {\pi \left( {r_{{\text{b}}} + a} \right)} }} $$

where $K_{IC}$ is the fracture toughness of the rock.

### Theoretical analysis of the new cartridge structure pre-cracking blast rock

**Figure 3:**
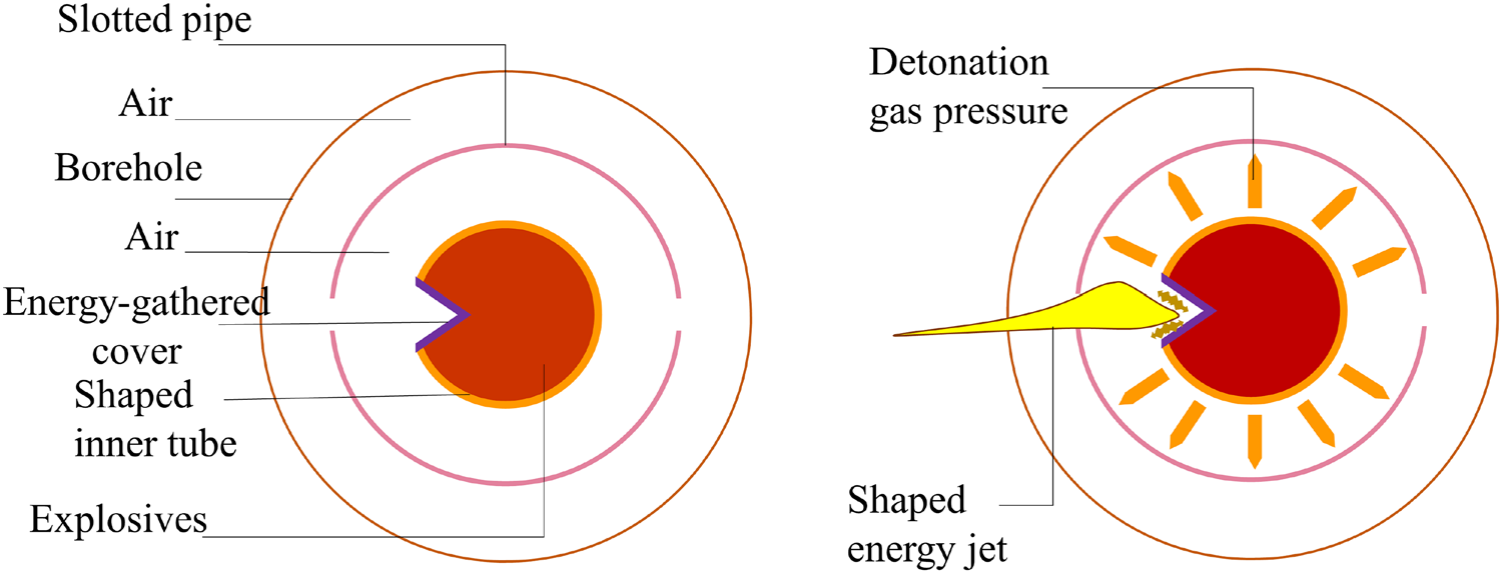
New cartridge structure blasting model.

The essence of the new cartridge structure discussed in this study is the combination of the slotted structure and the shaped charge structure. Its blasting model is depicted in Fig. 3. During detonation, the shaped energy cover forms an energy jet under the pressure of the internal explosive. This jet penetrates the rock mass through the gap in the slotted pipe. Once the initial crack forms in the hole wall, it expands preferentially under the combined effects of the detonation wave and explosive gas. This process ultimately results in directional cracking in the focused direction. The action mechanism of this new charge structure is derived from the mechanisms of both the slotted cartridge and shaped charge blasting.

In the blasting process of concrete using the new charge structure, for concrete in the test with a specific fracture toughness $K_{IC}$ and the radius of the borehole $r_{b}$, the blast gas pressure on one side of the new charge structure without a shaped hood is P1, and the explosion gas pressure P2 on the other side; therefore, the explosion gas pressure has the following expression:

5 $$ P_{1} F\sqrt {\pi (r_{b} + a)} > K_{IC} $$

6 $$ P_{2} F\sqrt {\pi (r_{{\text{b}}} + a)} + \sigma_{\theta } \sqrt {\pi a} > K_{IC} $$

7 $$ F = F[(r_{b} + a)/r_{b} ] $$

Due to the fracture toughness $K_{IC}$, the radius $r_{b}$ of the borehole is fixed, for Eq. (7) F is determined by the length $a$ of the radial fracture at the current moment; for Eq. (5) P1 is defined by the length $a$ of the radial fracture at the current moment; for Eq. (6) P2 is identified by the length $a$ and ${\sigma }_{\theta }$ of the radial fracture at the current moment, because ${\sigma }_{\theta }$ cannot be negative, if it is assumed that the length $a$ of the radial fracture at the current moment is the same length in both directions, then:

8 $$ {\text{P1 > P2}} $$

From Eq. (8), it is inferred that when the new type of medicine bag fractures the concrete, if P1 = P2, the cracking effect in the direction of the shaped mask surpasses that in the direction of the condensing mask. The new cartridge structure combines the features of the shaped charge and the slotted cartridge. In this study, the shaped energy mask and the incision are aligned horizontally. The initial guide crack in the direction of the energy-gathering hood is significantly larger than other minor cracks. As the high-pressure explosive gas enters this initial guide crack, the pressure in the energy concentration direction rises. Based on energy conservation, the explosive gas in the non-energy-gathering direction diminishes^40^. The shell of the slotted pipe situated outside the shaped energy cover serves a blocking role. This slotted pipe offers more protection for the rock mass in the non-energy-gathering direction than in the energy-gathering direction because the detonation wave directly induces a weaker air shock wave in the latter. Without any blocking casing, the high-pressure detonation gas quickly accumulates in the direction of the slit, amplifying energy concentration in that direction and accelerating it toward the borehole wall^41^. Hence, directional-shaped energy blasting enhances crack propagation in the combined energy-gathering direction while diminishing it in the non-energy-gathering direction.

## Trial of new cartridge structure

The shaped charge tubes employed in this experiment were PVC pipes from the same batch, with a wall thickness of 1 mm and an inner diameter of 18 mm. The cutting pipe's wall thickness was 2 mm, its cutting slit width was 2 mm, the cut length was 8 cm, and its inner diameter was 26 mm. The decoupling coefficient was 1.78. Parameters such as the control blasting environment, the borehole's aperture and depth, filling method, instrument and equipment design, the triangular hood's size, and the wall thickness and material remained consistent. All explosives used were from the same batch of No. 2 rock emulsion explosives. The linear charge density ranged from 1 to 1.25 g/cm^3^, the charge diameter was 13 mm, and the explosive configuration comprised a unilateral-shaped charge structure and a bilateral symmetrical slotted cartridge. Every charge was detonated using a standard No. 8 detonator. The first four were concrete tests to determine the dosage and length of the detonating cable; the last three experiments studied the influence of different slotted pipe materials on the pre-splitting blasting effect. In order to observe the blasting effect after parameter adjustment, the explosion and the formal tests were combined into one model test. The parameters were as follows: the dosage of emulsified explosive was 10 g, the detonating cord was 10 cm, and the combination of aluminum slotted pipe and PVC-shaped energy pipe was used as a double-layer constraint. Tables 1, 2, 3, 4, 5, 6, 7 is about the data of some experimental parameters.

**Table 1:** Test specific parameters.

Borehole diameter/mm	Borehole depth/cm	Stemming length/cm	Charge weight/g	Linear charge density/(g/cm^3^)	Decoupling coefficient	Slot length/cm	Slot width/mm
32	30	20	Determine the quantity based on trial blasting tests	1–1.25	1.78	8	2

**Table 2:** Relevant material parameters of the explosive.

Detonation velocity/(m/s)	Explosive density/(g/cm^3^)	Blasting pressure/Gpa
4300	1150	3.43

**Table 3:** Relevant material parameters of the copper charge liner.

Density/(g/cm^3^)	Thickness/mm	Shaped charge angle/°
8.96	0.1	60

**Table 4:** Relevant material parameters of the polyvinyl chloride tube.

Density/(kg/m^3^)	Elastic modulus/GPa	Poisson's ratio	Yield strength σ y/MPa	Wall thickness/mm	Inner diameter/mm
1300	3	0.25	22	1	18

**Table 5:** Relevant material parameters of the polyvinyl chloride slotted tube.

Density/(kg/m^3^)	Yield stress/MPa	Poisson's ratio	Elastic modulus/GPa	Tangent modulus/GPa	Wall thickness/mm	Inner diameter/mm
1300	30	0.38	3.7	0.45	2	26

**Table 6:** Relevant material parameters of the aluminum slotted tube.

Density/(kg/m^3^)	Elastic modulus/GPa	Poisson's ratio	Hardness	Yield strain rate/(1/s)	Plastic strain rate /(1/s)	Wall thickness/mm	Inner diameter/mm
2710	69	0.33	0.598	6500	4	2	26

**Table 7:** Relevant material parameters of the kraft paper slotted tube.

Density/(g/cm^3^)	Wall thickness/mm	Inner diameter/mm
1.64	2	26

### Fabrication of concrete test blocks

**Figure 4:**
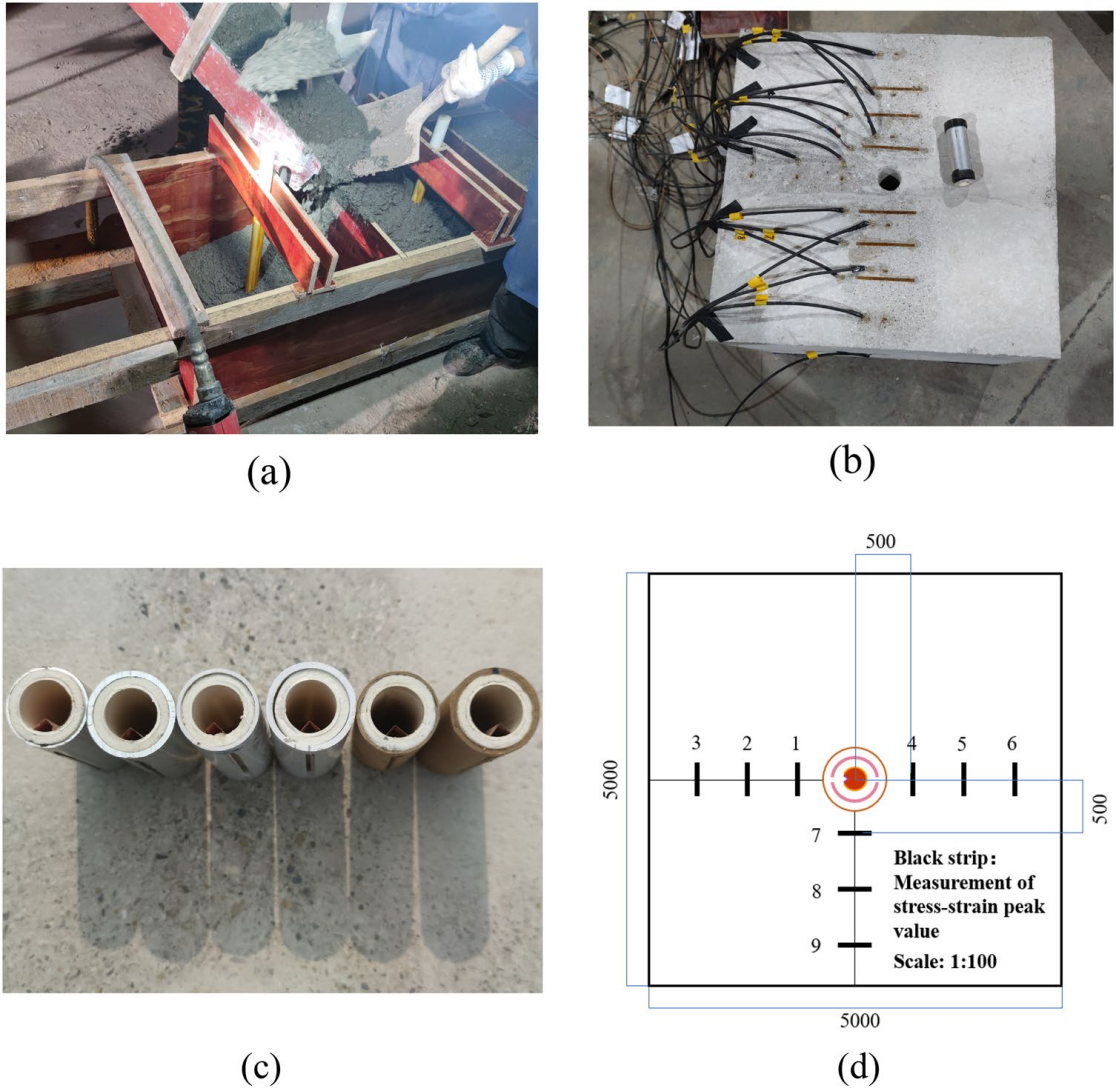
Concrete test block production. (**a**) Concrete in pouring. (**b**) The specimen after attaching the strain gauge. (**c**) Tubes with different slitted tube materials. (**d**) Schematic diagram of the point arrangement of strain gauges.

The C30 strength commercial concrete for this study was molded into six concrete blocks, each with dimensions of 50 × 50 × 50 cm. The designated borehole had a diameter of 32 mm and a depth of 30 cm. In order to meet the strength of the specimen, the concrete test block must be cured for 28 days, and to test the crack growth state around the borehole, a strain gauge is deployed around the borehole, as illustrated in Fig. 4.

### Introduction to test instruments

**Figure 5:**
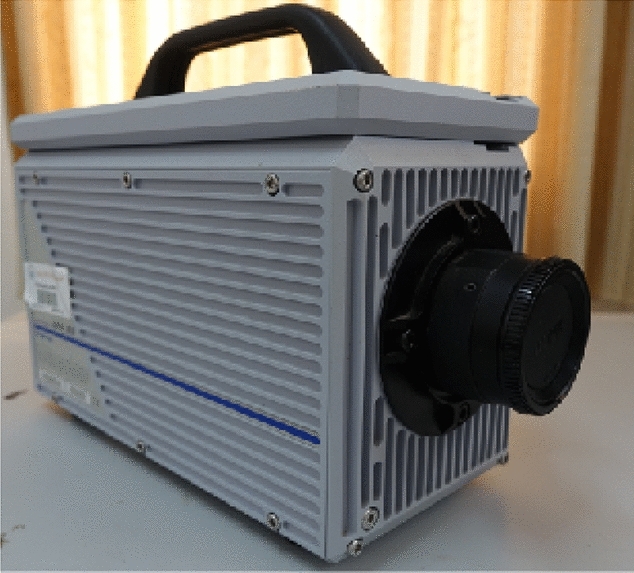
High-speed camera.

**Figure 6:**
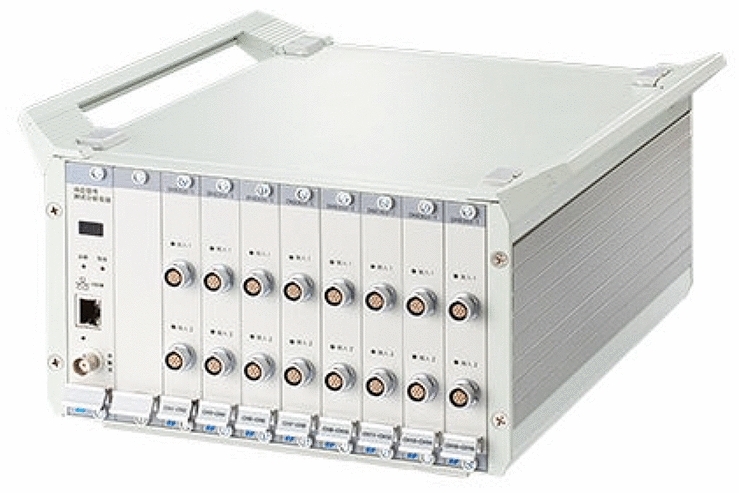
DH5960 working system.

This study employed an APX-RS model high-speed camera and a DH5960 and DHDAS5939 hyperdynamic signal testing and analysis system produced by the Jiangsu Donghua Test. The high-speed camera is depicted in Fig. 5, while the DH5960 hyperdynamic signal testing and analysis system is shown in Fig. 6.

### Concrete test process and result analysis

**Figure 7:**
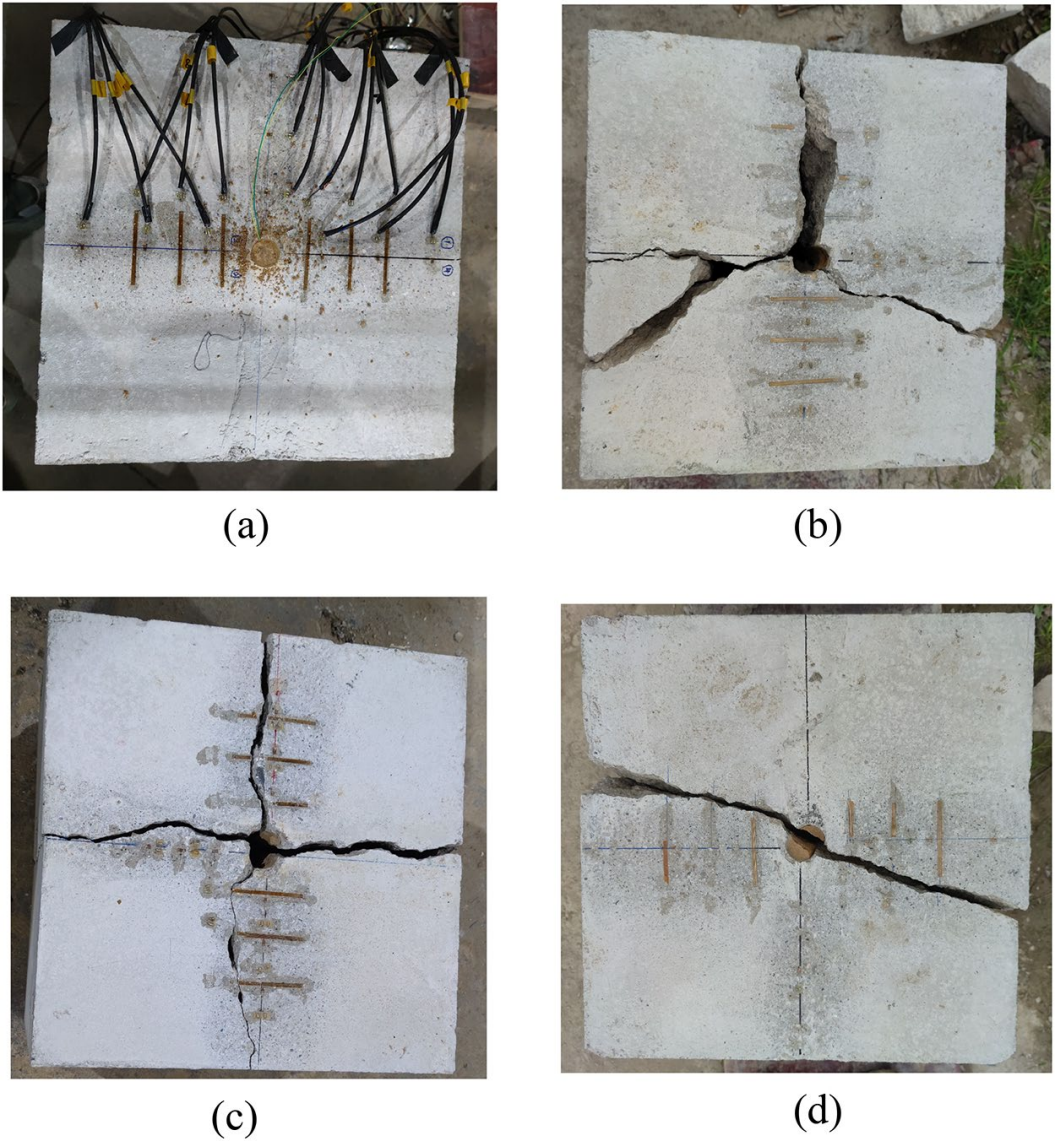
Effect diagram of test explosion test. (**a**) Method test (Explosion rejection). (**b**) Modality test (the first time did not burst, and the second time cracked). (**c**) Method test (first unburst, second punch, third cracking). (**d**) Method test (one detonation).

Four concrete samples underwent testing, with the blasting charge amounts determined by the concrete's crack propagation post-explosion. The charge and slotted pipe materials were as follows: (a) 10 g emulsion explosive, an aluminum slotted pipe, and PVC-shaped pipe as a double-layer constraint; (b) 10 g emulsion explosive with an 8 cm detonating cord, and PVC slotted pipe and PVC-shaped energy pipe as a double-layer constraint; (c) 8 g emulsion explosive with a 10 cm detonating cord, combined with kraft paper slotted pipe and PVC shaped energy tube as a double-layer constraint; (d) 10 g emulsion explosive, a 10 cm detonating cord, and an aluminum slotted pipe and PVC shaped energy pipe as a double-layer constraint. For this experiment, dry loess served as the filler, and the plugging loess mass (320 g ± 1 g) and plugging length (20 cm) were kept consistent. The explosion's outcome in concrete is presented in Fig. 7.

Based on the above figure, the A test experienced rejection. Following an investigation, the research team concluded that the charge structure and small diameter package can potentially compromise the detonation performance of the emulsion explosive. Hence, the addition of a detonating cord can enhance the detonation capacity.

The initial detonation using method B was successful without cracking the concrete. Subsequent hole treatment occurred after adequate pressure relief. In order to ensure the test's safety, the same quantity of explosive was used during the second attempt, which successfully cracked the concrete. The concrete module required two blasts to crack without disintegrating. Both blasts employed a detonating cord to enhance detonation efficiency. This indicates that the detonation of emulsion explosives is not linear, and a mere increase or decrease of 1–2 g of emulsion explosives might not significantly influence the outcome. However, increasing the length of the detonating cord can enhance the detonation capacity. After the end of two blasting, there was a crack in the combined energy-gathering direction and two cracks in the non-energy-gathering direction. Based on the expected results from method B, cracks should have appeared in the combined energy-gathering direction and the single energy-gathering direction, with minimal or no cracks in the non-energy-gathering direction. The results deviated from these expectations, potentially because concrete is nearly homogeneous. In the non-energy-gathering direction, primary cracks can objectively exist. Even if no visible cracks appear after the initial explosion, the inside of the concrete can sustain damage, the extent of which remains unidentified. Therefore, after the second blasting, damage was evident in the non-energy-gathering direction, while no cracks manifested in the energy-gathering direction. However, the combined energy-gathering direction did exhibit a directional crack effect.

The first detonation using method C was successful, but the concrete remained intact. A second attempt led to damage to the hole wall. In order to address this, the hole wall was grooved to increase the punching resistance, and by the third attempt, cracking was achieved. The concrete module cracked without disintegrating after three blasts. It is deduced that the packing methods of tests B and C were consistent in terms of the quality of the backfill loess. Before each initial test, the reserved borehole was inspected endoscopically and showed no damage. However, when the explosive charge for test C was decreased and the detonating cord length increased, the hole wall sustained damage. This confirmed that enhancing the detonation capability required an increased detonating cord, especially when using a less explosive amount. The overall trial emphasized the external slotted pipe's material constraints, and the explosion cartridges varied in their constraints. This meant the amount of explosive required to crack the concrete without disintegration varied. Therefore, the explosive amount and detonation cord length adjustments were deemed feasible. Following the blast, there was a crack in the combined energy-gathering direction, one in the single energy-gathering direction, and two in the non-energy-gathering direction. The expected results for test C suggested that cracks should appear in the combined energy-gathering and single energy-gathering directions, with few or no cracks in the non-energy-gathering direction. The actual outcomes did not fully align with these expectations. Given that the concrete was blasted three times before cracking, its internal damage can be intricate and undefined, making variations in crack patterns understandable.

The concrete cracked successfully after a single detonation using the D method test. Although the concrete cracked once, the two resultant pieces were projected from the center to the edge of the explosion tower, covering a distance of at least 4 m (the explosion tower has a diameter of 8 m). This indicates that the dosage was slightly excessive and should be reduced to ensure safety. The cracking largely conformed to the anticipated design direction; only a single penetrating crack appeared in the combined energy-gathering and the single energy-gathering directions, while the non-energy-gathering direction remained intact without any cracks. An analysis of the profile of the post-explosion concrete blocks suggests that a structural shift in the concrete was highly unlikely to be the cause of any angular deviation. The charge structure was probably slightly misaligned from its intended position when loaded into the borehole.

Accordingly, based on the dosages employed in these four tests, it is inferred that variations in the shell constraints of the charge structure lead to different optimal dosages for cracking the concrete without causing it to disintegrate. It is, therefore, recommended that distinct dosages be used corresponding to different structural constraints to better account for the variability in charge structure. Empirical data indicated that under the constraints of the aluminum slotted pipe material, the optimal dosage is 8.2 g with a 13 cm detonating cord. Under the constraints of the kraft paper slotted material and the PVC slotted material, the dosage should be 10 g with a 15 cm detonating cord. This research further explored the effects of different slotted pipe materials on pre-splitting blasting outcomes.

## Analysis of test results

### Explosive crack distribution characteristics

**Figure 8:**
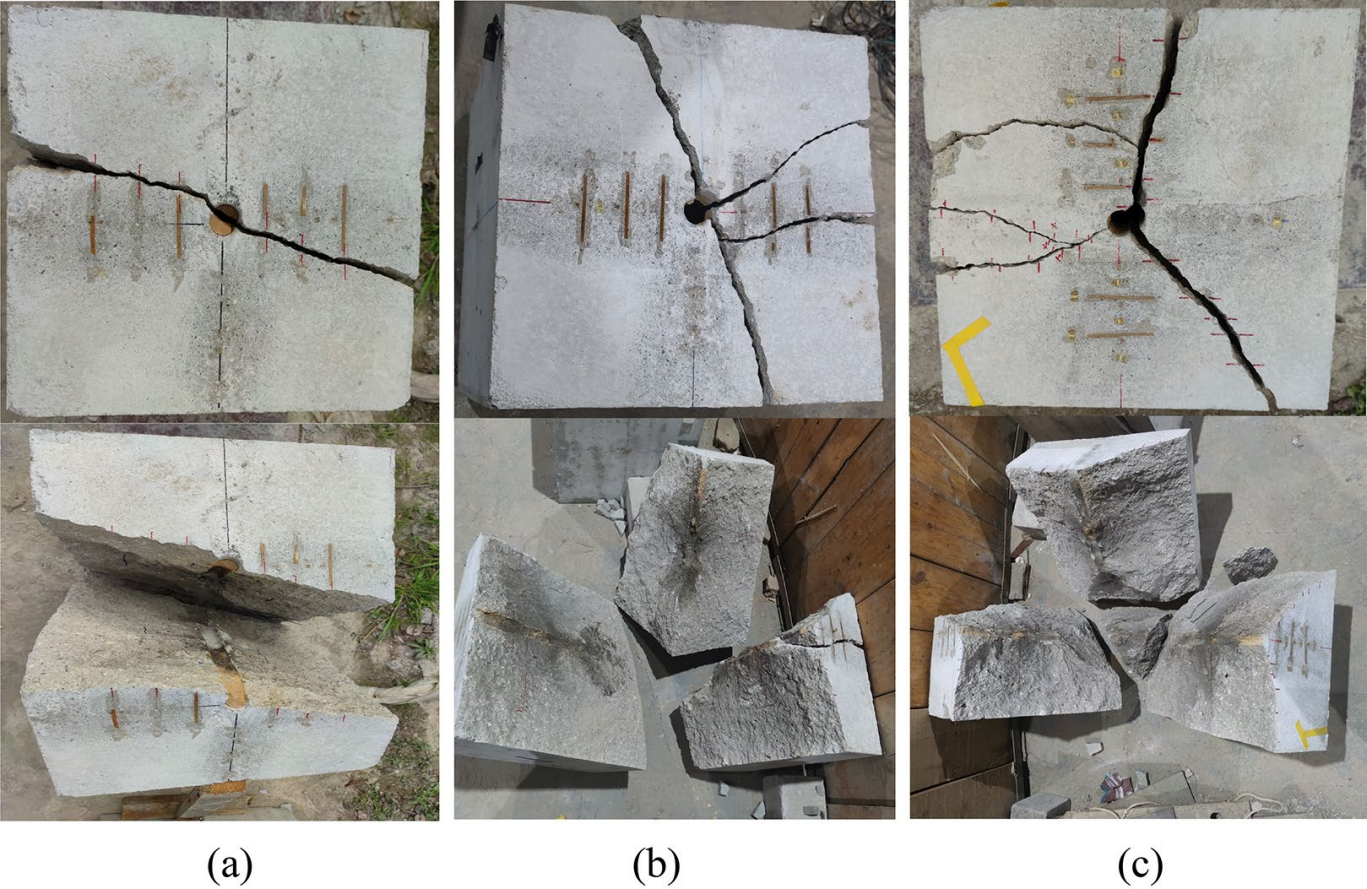
Post-explosion diagram of different materials of slit pipe. (**a**) Aluminum cut seam material medicine bag burst after the picture. (**b**) Kraft paper cutting material medicine bag burst after the photo. (**c**) PVC cut material after the explosion of the medicine pack.

The post-explosion visual data is presented in Table 8. From the standpoint of concrete crack width in the combined energy-gathering direction, the average crack width for the aluminum material was 1.17 cm, for the kraft paper material, it was 0.92 cm, and for the PVC material, it was 1.53 cm. Meanwhile, in the single energy-gathering direction observation, the average crack width for the aluminum material was 1 cm; for the kraft paper material, it was 0 cm, and for the PVC material, it was 1.47 cm. In the non-energy-gathering direction measurement, the average crack width for the aluminum material was 0 cm; for the kraft paper material, it was 0.88 cm, and for the PVC material, it was 0.5 cm. When comparing the concrete crack offset angle, using the center of the concrete as a reference perpendicular line, the offset of the combined energy-gathering direction is as follows: 10.6° for the aluminum material, 26.3° for the kraft paper, and 2.6° for the PVC material. Regarding the single energy-gathering direction, the deviation for the aluminum material was 11.7°, for the kraft material, it was 0°, and for the PVC material, it was 23.5°. In the non-energy-gathering direction, the deviations were 0° for the aluminum material, 18.2° for the kraft material, and 8.2° for the PVC material. From the perspective of explosive penetration distance in the combined energy-gathering direction, the distances were 16.3 cm for aluminum materials, 21 cm for kraft paper, and 21. 7 cm for PVC materials. In the single energy-gathering direction measurements, the distances were 12. 75 cm for aluminum materials, 0 cm for kraft paper, and 14. 75 cm for PVC materials. In the non-energy-gathering direction measurement, the distances were 0 cm for aluminum materials, 18.15 cm for kraft paper, and 12. 95 cm for PVC materials. Considering the filling residual distance, the values were 10.5 cm for the aluminum material, 11.73 cm for kraft paper, and 11.97 cm for the PVC material. The post-explosion effects of the slotted sleeves of different materials are depicted in Fig. 8. Based on a comparative analysis of the borehole post-explosion, the integrity of the concrete borehole's inner wall after the explosion is best preserved with the new aluminum slotted pipe material compared to the other two materials.

**Table 8:** Data table of intuitive data after the explosion.

Judging indicators	Direction	Model block serial number		
		#1	#2	#3
Crack width (cm)	The combined energy-gathering direction	1.17	0.92	1.53
	The single energy-gathering direction	1.00	0	1.47
	The non-energy-gathering direction	0	0.88	0.5
Cracking angle (°)	The combined energy-gathering direction	10.6	26.3	2.6
	The single energy-gathering direction	11.7	0	23.5
	The non-energy-gathering direction	0	18.2	8.2
Penetration distance (cm)	The combined energy-gathering direction	16.3	21	21.7
	The single energy-gathering direction	12.75	0	14.75
	The non-energy-gathering direction	0	18.15	12.95
Stuffing residual distance (cm)		10.5	11.73	11.97
Charge residual situation		A slit sleeve is present	There is no left	Half of the slotted pipe

From this comparative analysis, it can be inferred that when examining the crack width, explosive intrusion distance, and directionality of the explosive energy of the slotted casing of the three materials, the combined energy-gathering direction has a more pronounced effect than either the single energy-gathering direction or the non-energy-gathering direction. The aluminum slit casing shows the most effective directional fracturing, with minimal damage to the borehole wall post-explosion. The kraft paper material ranks second in directional fracturing, but its non-energy-gathering directional protection is weak. The PVC material is the least effective in directional fracturing of the slotted charge.

### Blasting energy successive release characteristics

**Figure 9:**
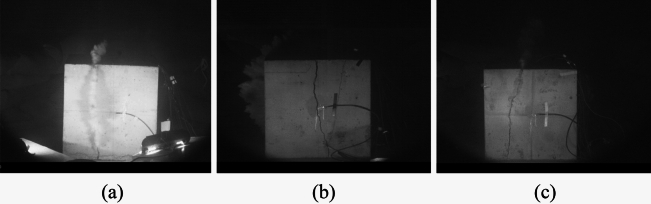
High-speed camera imaging map. (**a**) Aluminum cut seam material medicine bag high-speed camera imaging image. (**b**) Kraft paper cut and sewn material, medicine bag, high-speed camera imaging drawing. (**c**) PVC cutting material medicine bag high-speed camera imaging image.

The observation of crack propagation in both the combined energy-gathering direction and the non-energy-gathering direction was made using a high-speed camera, allowing for the deduction of the primary overflow direction of the explosive gas. As illustrated in Fig. 9a, when the slotted material is an aluminum pipe, the explosive gas overflows preferentially from the combined energy-gathering direction rather than the non-energy-gathering direction and the upper surface of the concrete. This suggests that the combined energy-gathering direction of the upper surface of the concrete tends to crack before the non-energy-gathering direction. In Fig. 9b, when the slotted material is kraft paper, cracks manifest on the concrete sidewall in the combined energy-gathering direction. Nevertheless, the explosive gas predominantly overflows from the non-energy-gathering direction, implying that the non-energy-gathering direction cracks before the combined energy-gathering direction. In Fig. 9c, when the slotted material is a PVC pipe, the explosive gas initially overflows from the upper surface of the concrete before emanating from the side wall in the combined energy-gathering direction. This indicates that the combined energy-gathering direction tends to crack before the non-energy-gathering direction.

From the above analysis, it can be seen that the slit material in which the explosive gas overflows preferentially in the combined energy gathering direction and cracks preferentially in the combined energy gathering direction is aluminum. Combined with the analysis of the explosion crack distribution characteristics, the charge directional cracking of aluminum slit casing The effect is good. The wall of the blast hole has not been pulverized and is basically intact after the explosion. This shows that the aluminum slit tube has a good energy guidance and concentration effect. When the slit material is kraft paper, considering the high temperature and high pressure released instantly by the explosive explosion, It far exceeds the binding capacity of the kraft paper tube. It is difficult for the explosion energy to be concentrated in the energy gathering direction, and the protective effect in the non-energy gathering direction is weak. As a result, the energy guidance and convergence of the slit tube made of kraft paper is not obvious. When the slit material is PVC, the explosion will occur. The gas first overflows from the upper surface of the concrete, and then overflows from the combined energy gathering direction. Combined with the analysis of the distribution characteristics of explosion cracks, it can be seen that the PVC slit pipe has more cracks. It can be seen that the energy guidance effect of the PVC slit pipe is better than that of the aluminum slit pipe. The seam tube is small and larger than the one made of kraft paper. Through the above comparative analysis, it can be concluded that the combined charge package made of aluminum slit tubes has a greater effect on the guidance and concentration of explosion energy than the combined charge package made of PVC slit tubes and the combined charge package made of kraft paper slit tubes.

### Analysis of strain test results

**Figure 10:**
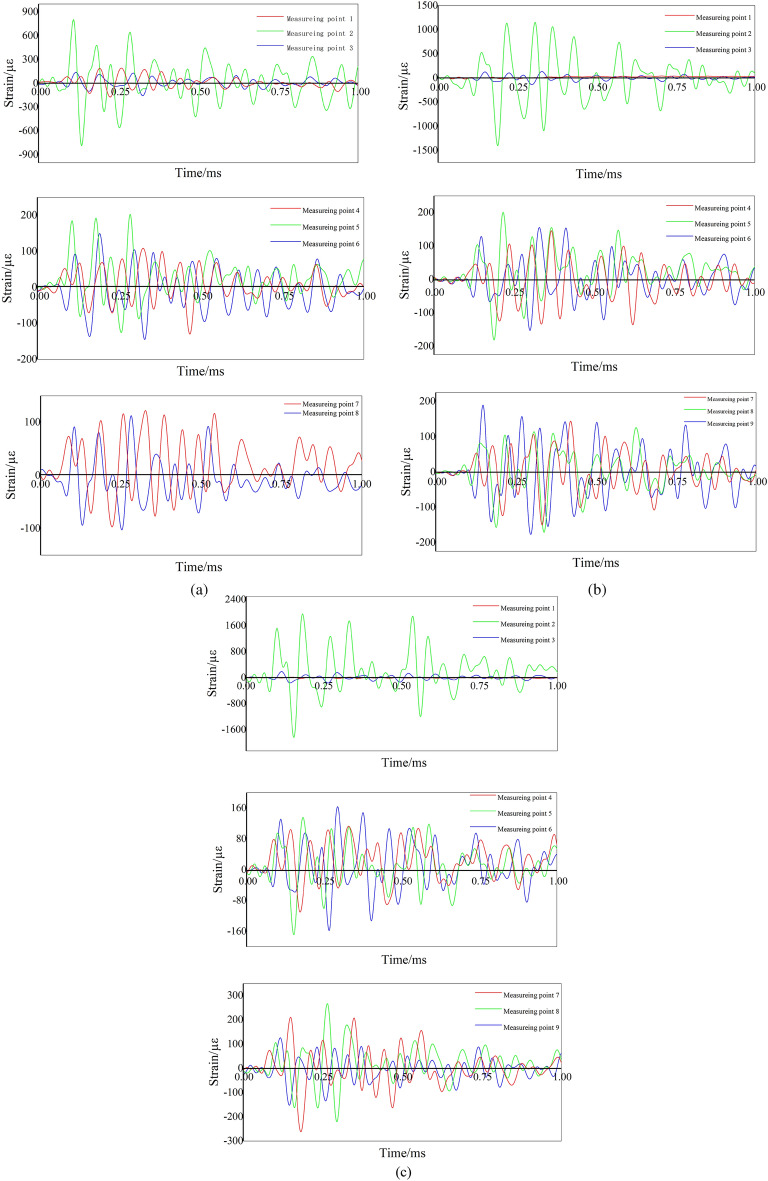
Strain waveforms of concrete with different numbers. (**a**) #1 Concrete strain waveform. (**b**) #2 Concrete strain waveform. (**c**) #3 Concrete strain waveform.

The strain test results, depicted in Fig. 10, reveal that strain values at measurement point 2 in the combined energy-gathering direction exceed those at measurement points 1 and 3. This discrepancy is likely due to the unique charge structure presented in this study, which results in a stress concentration at that point. The peak strain is reached approximately earlier on one side of the profiled hood than the stresses in the single energy-gathering and non-energy-gathering directions.

As observed in Fig. 10, with a wall thickness of 2 mm for the slotted pipe, measurement points 1–3 lie in the combined energy-gathering direction. The overall waveform exhibits an initial increase followed by a decrease. As per Table 9, the strain peak of the PVC slotted casing is the most pronounced, trailed by the kraft paper, while the aluminum slotted casing has the lowest strain peak. Despite this, the post-explosion effect diagram demonstrates that aluminum pipes' directional pre-cracking and non-energy-gathering directional deteriorating effects are superior. The disparity in the strain peak and explosion crack distribution form can be attributed to variations in dosage. Measurement points 4–6 are the single energy-gathering direction. The effect of the three materials is not much different, and there is no obvious rule. It is observed that the positive and negative peaks of the kraft paper slotted pipe material are slightly larger, and the main reason is that the binding effect of kraft paper is small, and the kraft paper will undergo greater deformation at the moment of explosion, resulting in a larger gap in the slotted seam. For measurement points 7–9 aligned in the non-energy-gathering direction, the absolute value of the positive and negative peaks are the least obvious when the aluminum slotted pipe bursts at measurement points 7 and 8. This suggests that aluminum provides the most effective retaining wall when used as the slotted pipe material.

**Table 9:** Measured results of peak blast strain for each model specimen.

Direction	Measurement point name	Model block serial number					
		#1		#2		#3	
		Peak positive strain (με)	Negative strain peaks (με)	Peak positive strain (με)	Negative strain peaks (με)	Peak positive strain (με)	Negative strain peaks (με)
The combined energy-gathering direction	1	191.78	− 172.60	53.27	− 21.06	17.06	− 25.95
	2	802.73	− 785.48	1236.14	− 1502.62	1957.56	− 1838.92
	3	138.10	− 155.61	133.69	− 137.43	191.24	− 162.33
The single energy− gathering direction	4	110.59	− 127.74	145.68	− 135.63	112.91	− 109.97
	5	202.94	− 125.51	200.58	− 181.14	135.77	− 168.44
	6	149.57	− 144.61	155.20	− 152.73	163.28	− 157.84
The non-energy-gathering direction	7	112.02	− 96.76	143.91	− 150.18	210.42	− 261.37
	8	111.44	− 103.50	125.57	− 170.67	266.66	− 220.60
	9			189.29	− 176.92	126.15	− 152.19

## Discussion

Based on the observed blasting effects on identical materials, the average crack width in the combined energy-gathering direction surpasses that in both the single energy-gathering and non-energy-gathering directions. Similarly, the invasion distance in the combined energy-gathering direction is greater than that in the single energy-gathering and the non-energy-gathering directions. When blasting the aluminum material cartridge, the cracking angle in the combined energy-gathering direction is below that in the single energy-gathering direction, while it is 0° in the non-energy-gathering direction. For kraft paper material cartridges bursting in the combined energy-gathering direction, the cracking angle is larger than in the non-energy-gathering direction, and it is 0° in the single energy-gathering direction. Considering the explosive's instantaneous release, which produces temperatures and pressures that vastly exceed the binding capacity of the kraft paper pipe, it becomes challenging to direct the explosion energy towards the energy-gathering direction, resulting in weaker protection in the non-energy-gathering direction. For PVC material, the cracking angle in the combined energy-gathering direction is less than those in the single energy-gathering and non-energy-gathering directions, and the crack direction is also shifted. There are three primary reasons for those: First, the shaped hood is only in the combined energy-gathering direction, and no metal jet directs the carved groove during the explosion in the other direction; Second, the first layer of the slotted pipe, made of PVC, offers limited restraint on the explosive energy; Third, the detonation wave passes through the air interval between the PVC pipe in the inner layer and the inner and outer layers of the PVC pipe, and after the energy is weakened, it penetrates the rock mass through the outer seam casing, and the explosion energy in the slotted direction is less than that of the conventional slotted cartridge. Thus, the combined effect of the slotted pipe and triangular-shaped cover in the combined energy-gathering direction provides the most effective directional fracturing, followed by the single energy-gathering direction, while the non-energy-gathering direction is the least effective.

**Figure 11:**
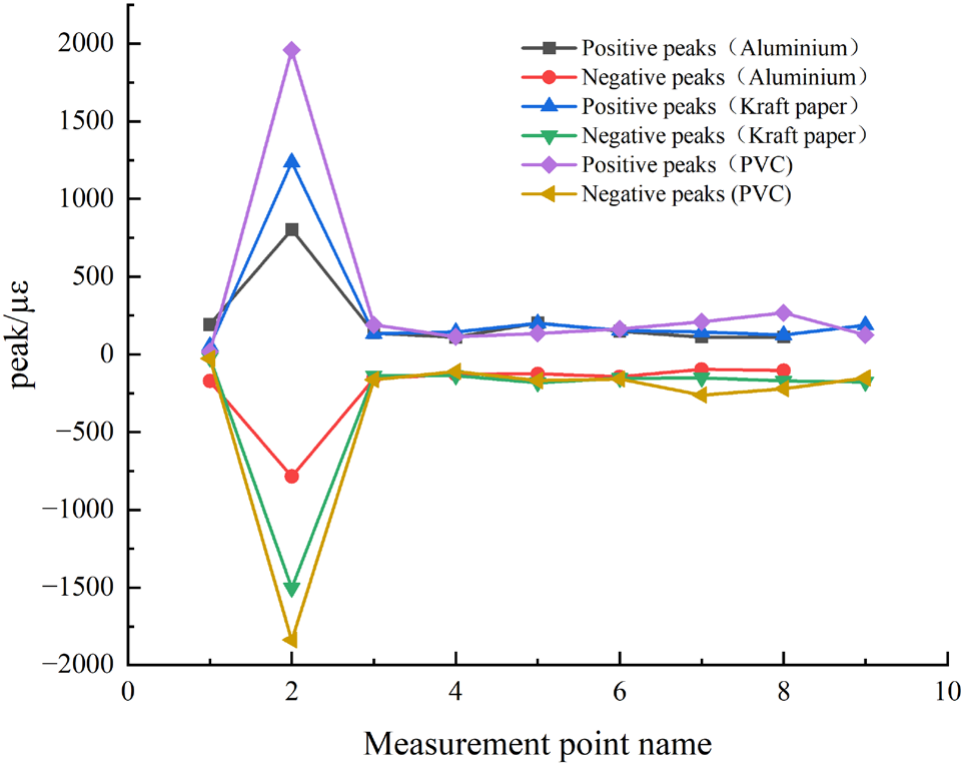
Variation in peak strain at each measurement point for different materials with 2 mm wall thickness.

In analyzing the blasting effects of different slotted pipe materials, the PVC material's directional cracking effect is superior to that of kraft paper when using equal amounts. However, PVC offers slightly inferior protection to the surrounding rock. The aluminum tube's directional pre-cracking and non-directional derogation effects are optimal. The strain peak and burst crack distribution patterns are minimal, primarily because of varied explosive quantities. Specifically, when using the same explosive amount, PVC and kraft paper cannot induce concrete cracking, but the derogation effect of aluminum surpasses that of PVC. Figure 11 illustrates the peak strain variation curve for 2 mm wall thickness materials.

## Conclusion

This study considers combinations of aluminum, kraft paper, and PVC material pipes with a wall thickness of 2 mm and a unilateral triangular concentrating hood to investigate the blasting effect of a novel explosive bag that combines a single-sided focusing hood and slit pipes made from various materials. It compares the blasting effects of these charge structures using theoretical analysis, explosion tests, and model tests. The main findings include:

1. By model explosion test, the explosive bag made from aluminum can crack concrete at the same dose. In contrast, those made from kraft paper and PVC cannot achieve this in a single attempt. This suggests that the binding capability of the aluminum slotted pipe material surpasses the other two materials. Using the aluminum slotted pipe explosive bag significantly reduces individual explosive consumption. Specifically, the dosage for the aluminum slotted pipe is 8.2 g, with a 13 cm detonating cord, while for the kraft paper and PVC slotted pipe, the dosage is 10 g with a 15 cm detonating cord.
2. When comparing different slotted pipe materials of the same wall thickness and considering the directional cracking and non-directional retaining wall conditions, the explosive crack distribution on the concentrated energy side, the sequence of blasting energy, and the strain waveform indicate the relative efficacy of each material. Based on the observed effects, the order is that aluminum surpasses PVC, which is superior to kraft paper.
3. The model test of various materials with identical wall thicknesses revealed differences in the behavior of explosion energy. In this test, the distribution of explosion cracks, the sequence of blasting energy, the consideration of strain waveforms were considered, and the recorded priority overflow direction of explosion gas for different materials using a high-speed camera was used, along with observing the actual post-explosion conditions, it is inferred that the aluminum slotted pipe has a more pronounced guiding and converging effect on explosion energy compared to the PVC slotted pipe and kraft paper slotted pipe. In addition, based on the comparison curve of positive and negative strain peaks in the three directions of the combined energy-gathering, single energy-gathering, and non-energy-gathering for different materials, and in light of the actual post-explosion conditions, it is evident that aluminum offers superior directional cracking effects compared to PVC and kraft paper.
4. The test employed C30-type concrete and utilized the combined shaped energy charging method, specifically the charging method of a double-sided slotted structure paired with a single-sided shaped hood. The slit structure guides the blasting energy, and the energy-gathering hood concentrates the blasting energy. After a comprehensive analysis, it is determined that the structure's cartridge provides a distinct and effective guiding influence on the blasting energy and exhibits the property of directional cracking. The new cartridge structure crafted from an aluminum slotted pipe can significantly decrease the required dosage amount while maintaining the desired blasting effect, offering a new blasting charge package structure for mining site applications similar to C30 concrete rock types.
5. The test results are clear, but the nature of the selected material is too different, making the results easier to speculate. However, each material test confirms that the directional rock blasting of the new cartridge structure of this experiment is feasible.

## Data Availability

All data, models, and code generated or used during the study appear in the submitted article. Because some data of this paper will be used in the next research plan of the research group, the data sets generated and/or analyzed in this study are not public, but reasonable requirements can be obtained from the corresponding authors.
